# Prebiotic chemistry: a review of nucleoside phosphorylation and polymerization

**DOI:** 10.1098/rsob.220234

**Published:** 2023-01-11

**Authors:** Xiaofan Guo, Songsen Fu, Jianxi Ying, Yufen Zhao

**Affiliations:** ^1^ Institute of Drug Discovery Technology, Ningbo University, Ningbo 315211, Zhejiang, People's Republic of China; ^2^ Department of Chemical Biology, College of Chemistry and Chemical Engineering, Xiamen University, Xiamen 361005, Fujian, People's Republic of China

**Keywords:** nucleoside phosphorylation, origin of life, polymerization, phosphates, minerals

## Abstract

The phosphorylation of nucleosides and their polymerization are crucial issues concerning the origin of life. The question of how these plausible chemical processes took place in the prebiotic Earth is still perplexing, despite several studies that have attempted to explain these prebiotic processes. The purpose of this article is to review these chemical reactions with respect to chemical evolution in the primeval Earth. Meanwhile, from our perspective, the chiral properties and selection of biomolecules should be considered in the prebiotic chemical origin of life, which may contribute to further research in this field to some extent.

## Graphical abstract

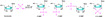

## 1. Introduction

When did life begin? Where did it begin? And how did it begin? These are some of the most important problems that modern science is attempting to answer. Various ideas have been proposed throughout this time, the most prominent of which are cosmic life theory [1], natural occurrence [2], chemical genesis hypothesis [3] and celestial impact theory. On this foundation of the chemical genesis hypothesis, the RNA world theory was established, which states that before the creation of DNA, the molecules of life appeared as RNA [4–6]. The production of RNA, from the synthesis of nitrogenous bases and ribose to the condensation of nucleosides and their subsequent phosphorylation and self-assembly, is thought to be crucial in understanding the origins of life [7]. The ‘RNA world’ concept suggests that a self-replicating molecule must be formed completely chemically in this process and that this macromolecule could be RNA [8,9].

A well-known statement by Nobel Laureate Professor Todd is ‘where there is life, there is phosphorus' [10]. Phosphorus is an essential element for the synthesis of various biological molecules and the execution of various biological functions. Phosphorylated molecules have a wide range of biological functions, and their role in life is very conserved, suggesting that they may have played important roles in the prebiotic Earth [11]. For example, phosphodiester serves as the structural skeleton of DNA and RNA, phospholipids are components of cell membranes, calcium phosphate tribasic is the main mineral component of bone and teeth, and high-energy phosphates can store biological energy. Thus, phosphorus is the central element of life [12].

As early as 1871, Darwin recognized the relevance of phosphate in protein synthesis in the origin of life [13,14]. Phosphorus also plays a significant role in the regulation of chemical evolution under prebiotic settings, and contributes to the synthesis of amino acids, nucleosides and other life-related raw materials, making it an indispensable component of prebiotic synthesis [15,16]. The majority of phosphorus on early Earth was in the form of water-insoluble minerals like apatite, which became the primary source of phosphorus for the Earth's phosphorus cycle [17]. In addition, reduced phosphorus species might constitute another phosphorus source in prebiotic period [18–20]. The ‘phosphate problem’ is exacerbated by the fact that most common phosphorus minerals are insoluble in water [21,22]. At the same time, the thermodynamically unfavourable processes of phosphorylation and polymerization of life molecules present a hurdle for primordial chemical synthesis.

Nucleosides are the basic components of nucleic acids (RNA and DNA) and are important biopolymers in life's chemistry [23]. The phosphorylation of primordial compounds like nucleosides, as well as their polymerization processes, is crucial for life's emergence [8,24–27], which have been investigated under a variety of settings, all of which use different phosphorylation sources and are constrained by reaction conditions [28,29]. This review provides an overview of nucleoside phosphorylation and polymerization in prebiotic conditions, summarizing the reaction processes of nucleoside phosphorylation and polymerization and providing fresh insights.

## 2. Prebiotic nucleoside phosphorylation

Although thermodynamically anhydrous circumstances were more favourable to nucleoside phosphorylation and polymerization in the early Earth environment [30], given its ubiquitous availability in primordial settings, water is still considered the most feasible solvent for the synthesis of the origin of life. As a result, a versatile and efficient phosphorylating agent that can phosphorylate a wide range of (pre-)biomolecules in water [31–33] and allow the formation of higher-order structures (oligonucleotides, peptides and liposomes) [34,35], is critical in the chemical environment of prebiotic systems.

### 2.1. Phosphorylation of nucleosides triggered by activators

**Figure 1.**
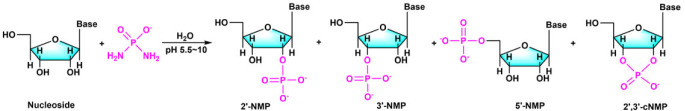
Nucleotide synthesis in neutral or alkaline aqueous solutions from DAP-activated nucleoside. Base = adenine, guanine, cytosine and uracil. Redrawn from [34].

A commonly used activator of nucleoside phosphorylation is diamidophosphate (DAP). The reaction of 0.1 M nucleosides and DAP in an aqueous solution at 50°C pH 5.5–10 produced 2′-NMP, 3′-NMP, 5′-NMP and 2′,3′-cNMP, while the addition of Zn^2+^ or Mg^2+^ or imidazole to the reaction system could accelerate the reaction or increase the phosphorylation of nucleosides [33,34] (figure 1). Further research has revealed that higher-order structures could be produced through oligomerization and self-assembly under the same phosphorylation reaction circumstances.

**Figure 2.**
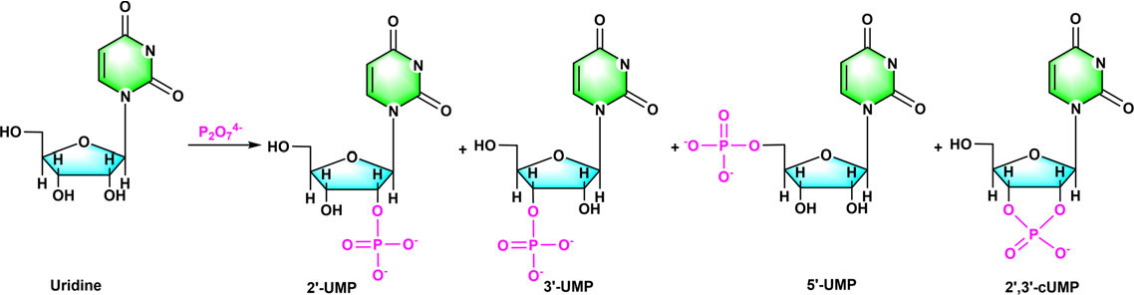
PPi-activated synthesis of nucleotides in an aqueous solution. Redrawn from [38].

Pyrophosphate (PPi) is a well-known prebiotic phosphorylation agent that is thought to be a ‘possible ancestor’ of the ATP molecule [36,37]. After the reaction of pyrophosphate with uridine (U) in an aqueous solution in the presence of white SiO_2_ sand, urea and Mg^2+^ ion in a simple prebiotic scenario simulating an evaporation/drying hot cell (60–75°C) generates a variety of phosphorylation products including 5′-UMP, 2′-UMP, 3′-UMP and cyclic 2′,3'-cUMP, as well as the dimerization products [38] (figure 2).

**Figure 3.**
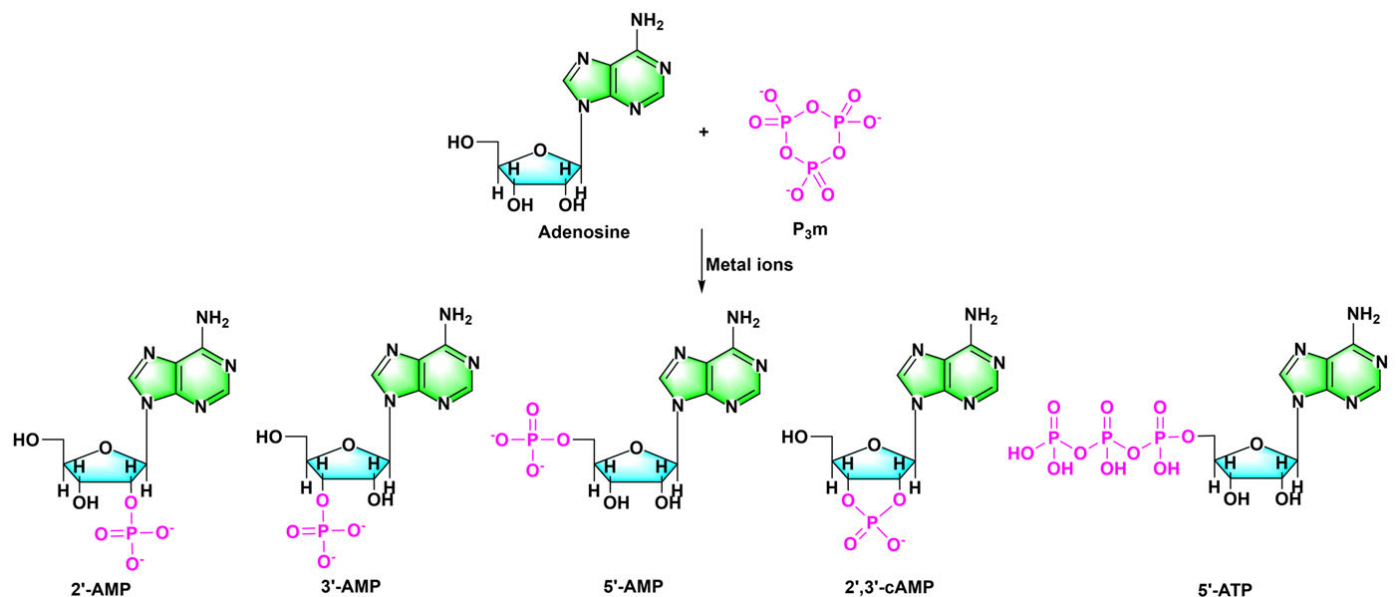
Nucleotide synthesis in aqueous solutions by P_3_m-activated nucleosides under various circumstances. Redrawn from [46].

Trimetaphosphate (P_3_m) is a water-soluble phosphate that enables the phosphorylation and polymerization of tiny biomolecules like amino acids and nucleotides [13,39,40]. The production of 2′-AMP and 3′-AMP by phosphorylating adenosine in the presence of sodium trimetaphosphate in an alkaline aqueous solution was first reported in 1969 [41]. At high pH and high temperature, all nucleosides are susceptible to phosphorylation by P_3_m, according to subsequent research [42,43]. At 90°C, a substantial amount of 5′-NMP is produced by the reaction of nucleoside, Ni(II), urea and P_3_m in the presence of boronate [44]. In addition, the reaction of P_3_m with adenosine in neutral aqueous solutions mediated by Mg^2+^ yields mostly 2′,3′-cNMP [45]. The reaction between 0.02 M adenosine and 0.2 M P_3_m in an aqueous solution in a wet and dry cycle (37°C, pH 7), catalysed by metal ions (particularly Ni(II)), yields a variety of phosphorylation products, including 5′-ATP, 5′-AMP, 3′-AMP, 2′-AMP and 2′,3′-cAMP [46–48]. It is worth noting that the 5′-ATP obtained by this method is considered to be very difficult to synthesize [39,49] (figure 3).

**Figure 4.**
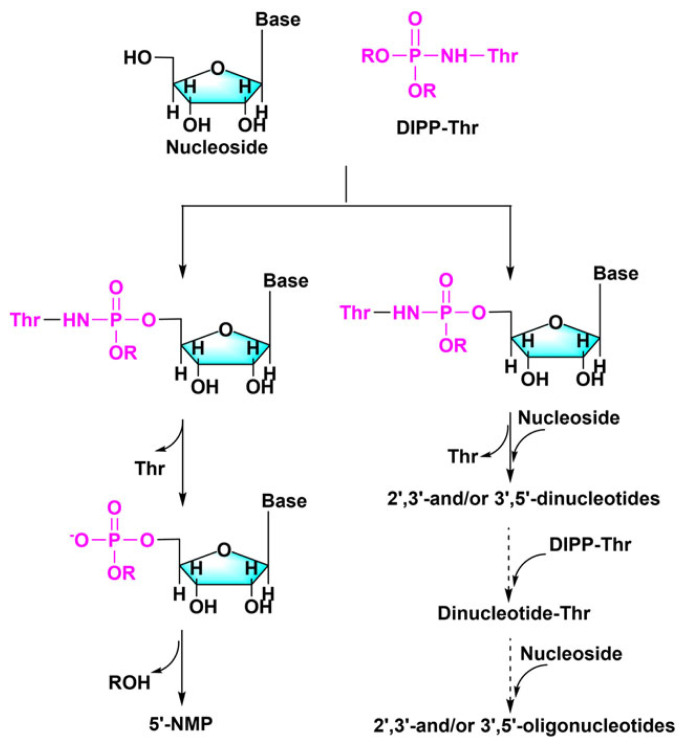
Mechanism by which N-phosphothreonine acts as an activator for the synthesis of nucleotides in an aqueous solution. Redrawn from [50].

It is even feasible to create both nucleotides and peptides by direct reaction between nucleosides and phosphorylated amino acids via intramolecular inter-activation between the phosphoryl and carboxyl groups [50,51]. Phosphorylated amino acids served as activators in this case. Take N-phosphothreonine as an example, the reaction of N-(O, O-diisopropyl) phosphothreonine (DIPPThr) and four nucleosides results in the formation of a phosphorylated oligonucleotides, with the major phosphorylation product being 5′-NMP (figure 4).

In addition to the foregoing, phosphorothioates [52,53], diiminosuccinonitrile (DISN) [54,55], adenine derivatives [56], and other activators or catalysts for nucleoside phosphorylation have been reported. Meanwhile, some superphosphates (branched phosphates) are able to phosphorylate nucleophiles such as amino acids and nucleosides [57], although this has yet to be investigated further because they are unstable in the presence of water.

### 2.2. Nucleoside phosphorylation using phosphorus-containing minerals

It has been established that on the Earth before the origin of life, the main sources of phosphorus are fully oxidized phosphates and reduced phosphorus species. The fully oxidized phosphates are most abundant species in phosphorus-containing minerals such as apatite which has long been considered the only significant source of phosphorus on Earth. However, the poor solubility and weak reactivity make them difficult to use in the prebiotic chemistry thus attract a lot of attention and research in response to this ‘phosphate problem’. Reduced phosphorus species, represented by schreibersite (Fe, Ni)_3_P, have now also been recognized as an important source of phosphorus in the early Earth. The reduced phosphorus compounds are mostly enriched in meteorites [18,58], seems to have existed in early Archean ocean [20], and also could be formed in extreme reduction conditions like lightning strike [19,59]. In general, current efforts mainly focus on exploring the utility of fully oxidized phosphates in the phosphorylation of nucleosides, whereas there are only individual cases about reduced phosphorus.

**Figure 5.**
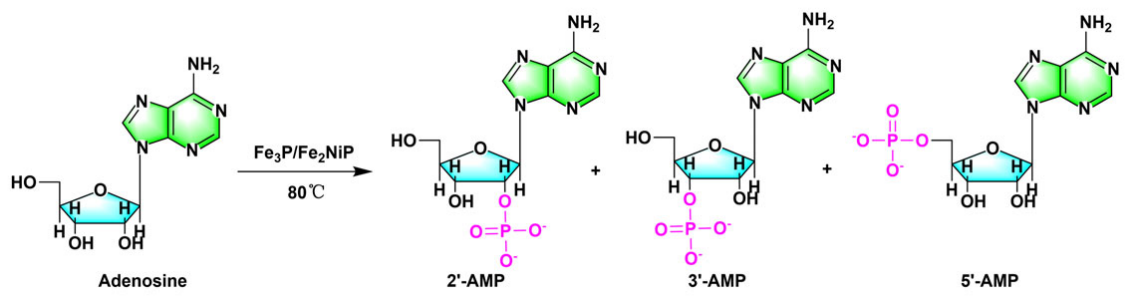
Nucleotide synthesis involving schreibersite (Fe, Ni)_3_P in aqueous solution (adenosine, in this example). Redrawn from [60].

It has been shown that synthetic schreibersite analogues (Fe_3_P and Fe_2_NiP) can react with adenosine or uridine to generate corresponding phosphorylation products by the simple mixing and mild heating of reactants (figure 5) [60]. Despite the lack of more cases, we believe that reduced phosphate minerals have non-negligible contributions to the phosphorylation of nucleosides in the early Earth. Indeed, experiments have shown that the reduced phosphorus minerals can be easily converted to water-soluble, highly reactive phosphorus-containing compounds that potentially serve as phosphorylation reagent for nucleoside phosphorylation [33,61,62].

**Figure 6.**
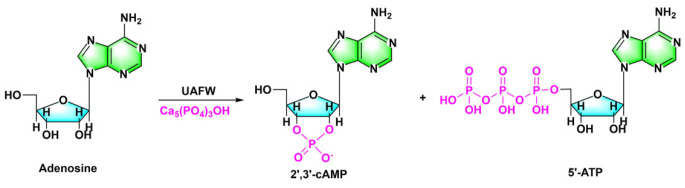
In the hemihydrate solvent UAFW, the reaction of Ca_5_(PO_4_)_3_OH with adenosine produces 5'-AMP and 2′,3'-cAMP. Redrawn from [63].

Ca_5_(PO_4_)_3_OH is typically a low-solubility mineral, but studies in recent years have shown that it could be dissolved in a semi-aqueous solvent UAFW which could improve the solubility of some insoluble phosphates [26,63,64]. After several days of reaction at 65 or 80°C, efficient phosphorylation of adenosine with hydroxyapatite Ca_5_(PO_4_)_3_OH in semi-aqueous solvent eutectic was observed, generating 5′-AMP and 2′,3′-cAMP (figure 6). Under these conditions, the corresponding oligomers were also recognized.

**Figure 7.**
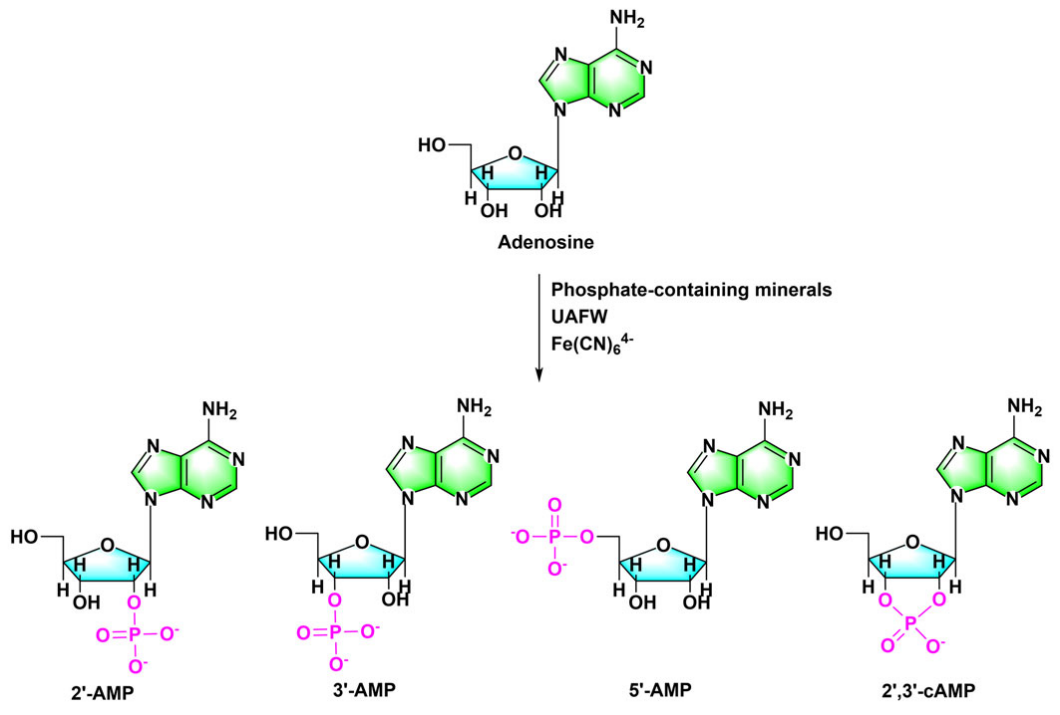
In the hemi-aqueous solvent UAFW, mechanisms by which cyanide and iron stimulate the interaction of phosphate with adenosine to form AMP. Redrawn from [65].

In addition cyanide and iron can also facilitate nucleoside phosphorylation by converting insoluble phosphate minerals in the ‘warm little pond’ into more soluble and reactive compounds [65–67]. Phosphorylation of adenosine in UAFW solvents was investigated using vivianite, iron phosphate, sodium phosphate monobasic, newberyite and hydroxyapatite as phosphate sources; all of these phosphate sources produced similar phosphorylation products dominated by 5′-AMP, 2′-AMP, and 3′-AMP, 2′,3′-cAMP (figure 7). The reaction between 5′-AMP and calcium phosphate also generates 5′-ADP and 5′-ATP in an aqueous solution in the presence of cyanate [49].

Formamide has been proposed as a significant component of nucleic acid formation [68]. Several phosphate minerals can act as phosphate donors for nucleosides in the presence of formamide, inducing phosphorylation of nucleosides [69,70]. By pretreating minerals with formamide (130°C, 72 h), then cooling to 90°C and adding nucleosides, a range of minerals with catalytic nucleoside phosphorylation activity were discovered, such as hydroxyapatite [71], libethenite ${\mathrm{Cu}}_{2}^{2+}({\mathrm{PO}}_{4})(\mathrm{OH})$, ludjibaite ${\mathrm{Cu}}_{5}^{2+}{\left({\mathrm{PO}}_{4}\right)}_{2}{\left(\mathrm{OH}\right)}_{4}$, reichenbachite ${\mathrm{Cu}}_{5}^{2+}{\left(\mathrm{PO}4\right)}_{2}{\left(\mathrm{OH}\right)}_{4}$, cornetite ${\mathrm{Cu}}_{3}^{2+}({\mathrm{PO}}_{4}){\left(\mathrm{OH}\right)}_{3}$ and hydroxylapatite Ca_5_(PO_4_)_3_OH [72], etc. 2′,3′-cNMP is the more stable phosphorylation product in the formamide reaction system. In addition, adding borate (Na_2_B_4_O_7_ · 10H_2_O) to an adenosine-inorganic phosphate-pyrophosphate reaction system in the presence of formamide and incubating at 90°C yielded the only phosphorylation product −5′-AMP [26,73].

Various approaches have been tried to overcome above two types of nucleoside phosphorylation (activator/ mineral-mediated) in water problems. One of which is heating orthophosphates (or minerals), nucleosides and condensates (with/without minerals as catalysts) to dryness or wet and dry cycles [25,46,66,74–78]. The approach is effective for prebiotic phosphorylation, and a comparable environment may have prevailed on the early Earth. Another interesting method is to add extra compounds to some of the above reaction systems to aid catalytic nucleoside phosphorylation. These additional substances do not play a dominant role, but rather serve as a facilitator. The addition of urea and ammonium chloride [78], ammonium oxalate [79] and other ammonium salts to a reaction mixture of nucleoside and neutral or basic phosphate, for example, allows for high yields of nucleoside phosphorylation. Metal ions, like Mg^2+^, could help to enhance nucleoside phosphorylation [45,80,81]. Furthermore, Mg^2+^ also aids in the conversion of insoluble phosphates (apatites) into more soluble minerals (struvite, MgNH_4_PO_4_·6H_2_O) [26]. As a result, it could have played a significant role in primordial chemical reactions. In addition, electrical discharges [75], lunar soil, ultraviolet sunlight (UV 254 nm), and vacuum ultraviolet light (VUV 145 nm) [82] are also effective at promoting nucleotide synthesis.

### 2.3. Phosphorylation of nucleosides in hypo-hydrous environments (aerosols, eutectic solvents)

Even though the water was the most appropriate solvent on the early Earth, phosphorylation was unfavourable to occur in aqueous conditions [83]. Many anhydrous reagents have been advocated to address this issue, including the various possibilities of nucleoside phosphorylation in an anhydrous environment [28,29,64,72,73,84]. Schoffstall postulated the prebiotic phosphorylation of nucleosides in formamide solvents as early as 1976 [28]. He also highlighted the alternate approaches afforded by phosphorylation in organic solvents in a series of groundbreaking studies [85–87].

**Figure 8.**
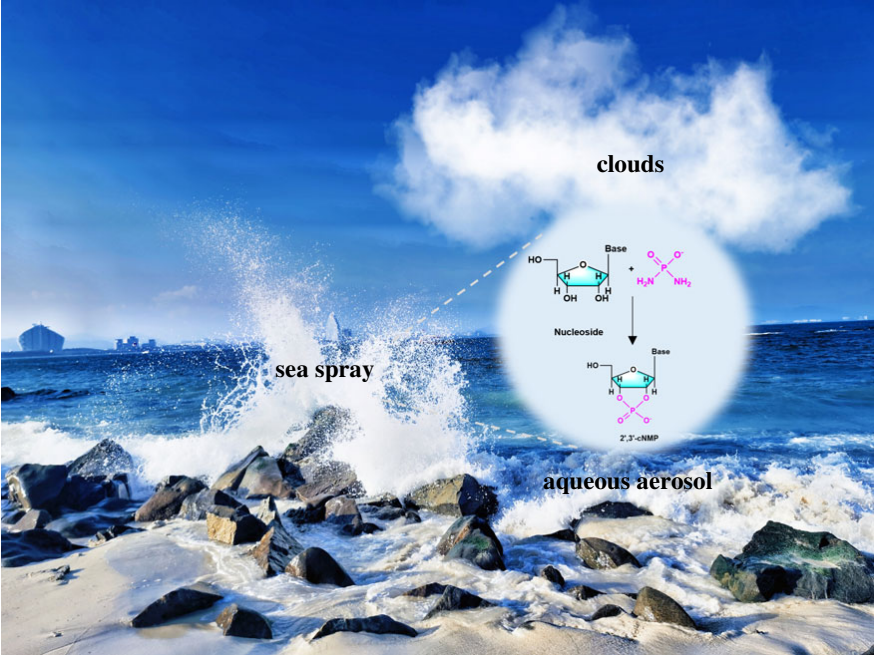
Natural aerosol reaction environment.

Although diamidophosphate (DAP) was used to phosphorylate several prebiotic compounds (nucleosides, amino acids and lipid precursors) under aqueous circumstances [88,89], the conversion rate was modest. Furthermore, it has been demonstrated that extending DAP phosphorylation to an aerosol environment (water/organic droplets generated at the water–air interface) boosts the rate of uridine phosphorylation to form 2′,3′-cUMP by more than 100-fold [90]. The water–air interface (microdroplets) may also be a useful model for understanding prebiotic phosphorylation because the early Earth was more than 90% covered by water, making water-containing atmospheric aerosols more abundant than they are now [91,92] (figure 8).

Phosphate can also be released from insoluble minerals by replacing the aqueous environment with eutectic and other low-water solvents [26,64,72]. For example, the use of the urea: choline chloride eutectic (DES) was proposed to increase phosphorylation of sugars and nucleosides at 60–70°C, in addition to facilitate Watson–Crick base pairing [93]. However, it is believed that the relevance of eutectic mixtures on the origin of life needs to be further explored [26,64,84,94], so it is briefly described here.

## 3. Polymerization of nucleotides

During the phosphorylation of nucleosides to create nucleotides, nucleotide oligomers can also be formed [34,38]. Even phosphorylated threonine oligopeptides can be produced directly under the catalysis of DIPP-Thr as shown in the right route of figure 4 [50]. Furthermore, many prior investigations have indicated that phosphorylation of prebiotic substrates in aqueous settings occurs more frequently in incompatible and unique environments, resulting in the formation of matching oligomers or higher order structures [95–102]. The oligomerization of nucleotides was first attempted by heating 2′/3′-NMP at 160°C, which resulted in a complicated mixture of very short oligonucleotides [103]. However, low yields and the ability to synthesize oligomers of limited length are common problems. To address these problems, a number of external activators [104,105] have been used to achieve longer oligonucleotide formation in sustained yields, with the following being the more effective ones.

### 3.1. Phosphorus imidazole-accelerated polymerization

**Figure 9.**
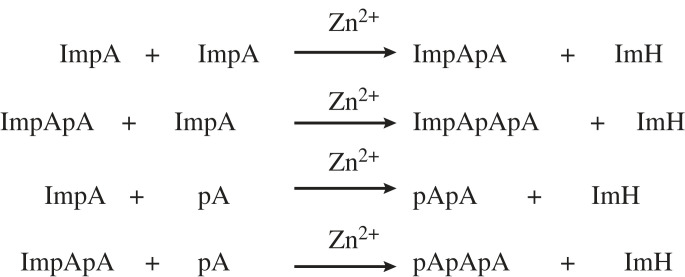
Representation of oligonucleotide production from adenosine S'-phosphorimidazolide (ImpA).

Under prebiotic conditions, the polymerization of nucleotides was investigated using phosphorimidazoles as reaction substrates. Adenosine S'-phosphorimidazolide (ImpA), as an example [106], underwent self-assembly to form oligonucleotides (figure 9). And studies of this polymerization process revealed that metal ions such as Pb^2+^, Zn^2+^ and Lu^3+^ accelerated the creation of pentamers, tetramers and trimers [107–110]. Uranyl ion ${\mathrm{UO}}_{2}^{2-}$ is the most effective among these metal ions, with the 2′,5′ linked phosphodiester bond dominating.

### 3.2. Clay mineral montmorillonite-promoted polymerization

**Figure 10.**
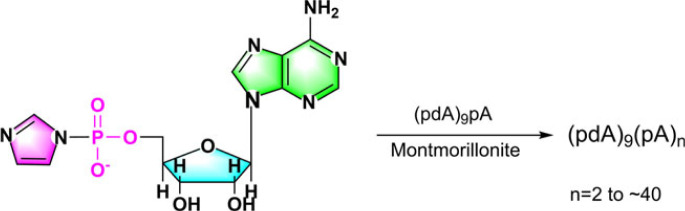
Prolongation of oligonucleotides by daily addition of the activating monomer ImpA in the presence of montmorillonite.

Montmorillonite, a clay mineral, is also a relatively effective catalyst [111–116] for oligomer formation even with dilute solutions of activated nucleotide substrates. Ferris *et al*. [115] investigated the oligomerization of nucleoside 5′-phosphate imidazoles and their associated activated nucleotides on the clay mineral montmorillonite (figure 10). In addition, subsequent research found: This mineral can help create up to 50-mer RNA by facilitating the reaction of mixed 2′,5′ and 3′,5′ phosphodiester-linked oligomers [116]. There is also a product-specific influence on the oligomerization reaction: adenosine-5′-phosphorimida-zolide on montmorillonite produces mostly 3′,5'-linked oligomers, whereas water produces mostly 2′,3'-linked oligomers [117,118].

### 3.3. Lipid-mediated polymerization

**Figure 11.**
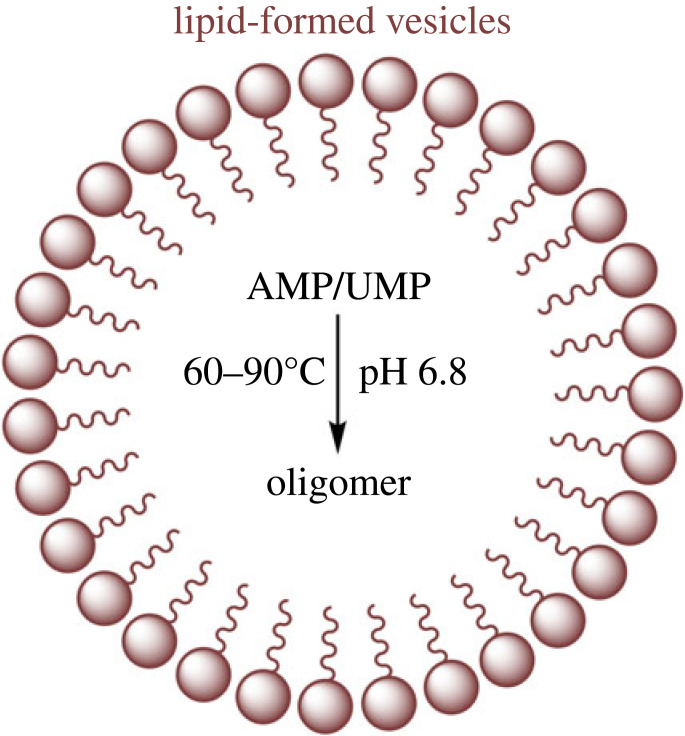
A model for nucleotide polymerization in a lipid-forming vesicle environment.

In addition, some scientists have demonstrated that RNA polymerization analogues can be synthesized non-enzymatically from single nucleotides in a lipid environment [119,120]. The experiments were carried out mainly by analysing the degree of polymerization of nucleotides in various lipid-forming vesicle environments (POPC (palmitoyl-oleoylphosphatidylcho-line), POPA (palmitoyl-oleoylphosphatidic acid) and LPC (lysophosphatidylcholine)), originally identified by nanopore analysis (a technique with single-molecule sensitivity), with polymerization lengths of up to 100-mer (figure 11).

### 3.4. Organoamine-accelerated polymerization

As early as 1971, Oro *et al*. reported the condensation of deoxythymidine-5′-phosphate using cyanamide as an activator [104]. In subsequent studies, oligomeric deoxynucleotides could also be synthesized in the presence of cyanamide and 4-amino-5-imidazole carbox-amide (AICA), but the yields were not high [121–124]. Therefore, the advantages of this type of activator over the three mentioned above in terms of yield and degree of nucleotide polymerization are not so obvious.

## 4. Conclusion and perspectives

Thanks to those tenacious practitioners, great progress has been accomplished in this field [125–127], who have researched the identification of fascinating abiotic syntheses. Nonetheless, due to the uncertainty of what chemicals may have been present on the early Earth and the lack of ‘chemical fossils’ to corroborate the postulated chemical pathways, there are still worrying uncertainties [128,129]. Although the early Earth's environment cannot be fully duplicated, it is felt that studying these questions, which have the potential to disclose the most fundamental features of life, is worthwhile. More discoveries and advancements will be achieved in the future as the chemistry of phosphorylation and polymerization of nucleosides under prebiotic conditions—a hot spot for research on the subject of life origins—is explored. Because organisms show chiral preferences for L-amino acids and D-nucleosides, it's possible that chirality had a role in biological evolution [130,131]. We believe that considering chirality will lead to a breakthrough in this field of study.

## Data Availability

This article has no additional data.
